# Thyroid shields and neck exposures in cephalometric radiography

**DOI:** 10.1186/1471-2342-6-6

**Published:** 2006-06-13

**Authors:** Philippe Hujoel, Lars Hollender, Anne-Marie Bollen, John D Young, Joana Cunha-Cruz, Molly McGee, Alex Grosso

**Affiliations:** 1 Department of Dental Public Health Sciences, School of Dentistry University of Washington, Seattle, USA; 2 Department of Epidemiology, School of Public Health University of Washington, Seattle, USA; 3 Radiology Clinic, Oral Medicine, School of Dentistry University of Washington, Seattle, USA; 4 Department of Orthodontics School of Dentistry University of Washington, Seattle, USA; 5 Environmental Health and Safety, Hall Health Center, University of Washington, Seattle, USA

## Abstract

**Background:**

The thyroid is among the more radiosensitive organs in the body. The goal of this study was twofold: (1) to evaluate age-related changes in what is exposed to ionizing radiation in the neck area, and (2) to assess thyroid shield presence in cephalometric radiographs

**Methods:**

Cephalometric radiographs at one academic setting were sampled and neck exposure was related to calendar year and patient's gender and age.

**Results:**

In the absence of shields, children have more vertebrae exposed than adults (p < 0.0001) and females have more neck tissue exposed inferior to the hyoid bone than males (p < 0.0001). The hyoid bone-porion distance increased with age (p <0.01). Thyroid shields were visible in 19% of the radiographs and depended strongly on the calendar year during which patient was seen (p-value <0.0001). Compared to adults, children were less likely to wear thyroid shields, particularly between 1973 and 1990 (1.8% versus 7.3% – p-value < 0.05) and between 2001 and 2003 (7.1% versus 42.9% – p-value < 0.05).

**Conclusion:**

In the absence of a thyroid shield, children have more neck structure exposed to radiation than adults. In agreement with other reports, thyroid shield utilization in this study was low, particularly in children.

## Background

Several radiosensitive organs such as the thyroid, the esophagus, and vertebral bone marrow are located in the neck tissues. The extent to which these organs are exposed to ionizing radiation under real-life conditions has not been reported and can vary due to person-to-person variability in location of thyroid position [1], due to the age-dependent caudal movement of the thyroid through puberty [2], due to clinician's request regarding what should be visualized on the radiograph [3], and due to differences in radiographic practices. There have been assertions that thyroid shields are commonly used in "modern" dentistry [4-6], but these assertions are inconsistent with survey information [7,8].

Current guidelines from the National Council on Radiation Protection and Measurements (NCRP) on thyroid collar utilization during cephalometric radiography are unclear. The NCRP reported that: "thyroid shielding shall be provided for children, and should be provided for adults, when it will not interfere with the exam" [9], and that 'no anatomical structures inferior to the hyoid should be visualized'[9]. Since thyroid shields may 'interfere' with the soft tissue examination below the chin and the cervical vertebrae, it is unclear whether current guidelines suggests that thyroid shields should be used with cephalometric radiography. The goal of this study was to assess two issues on cephalometric radiographs: (1) to evaluate anatomical characteristics of neck exposures in the absence of shielding, and (2) to estimate the prevalence of thyroid shield utilization.

## Methods

### Data abstraction

The Department of Orthodontics at the School of Dentistry of the University of Washington maintains approximately 3,500 historical records of individuals which were referred for orthodontic records between 1965 and 2005 and that were not seen on a regular basis at the time this study was conducted. The charts are classified alphabetically and a systematic sample of available charts present in the department was taken between July 2005 and November 2005. The term systematic sampling indicates that every 6^th^, then every 5^th^, chart was selected. Data were abstracted by a dental student (J.D.) and an investigator (P.H.). In case of uncertainties related to data abstraction, the radiologist on call was contacted to make decisions Data from the selected charts were abstracted using a standardized approach and included gender (male, female), date of birth (month and year), and the date and type of intra- or extra-oral radiographs. The current report focuses on the frontal and lateral cephalometric radiographs that were taken. When the records indicated that a cephalometric radiograph was taken, but it was not present in the chart, or it was not dated, or it was not of diagnostic quality, it was not included in the analysis. Approval for conducting the research was obtained from the Human Subject's Division at the University of Washington.

#### Anatomical neck characteristics

The anatomical characteristics were abstracted from the cephalometric radiographs in which no shielding (thyroid or apron) were observed. The anatomical information included the most inferior vertebrae, the position of the hyoid bone relative to the lower border of the X-ray, and the position of menton (the most inferior point on the symphysis of the mandible) relative to the lower border of the film (both measured perpendicularly from the lower border of the X-ray). The most inferior vertebra (C1-C7, T1-T2) on the film was considered to be exposed when any part of the ventral surface of the body of the vertebra was visible. Since the ear is in a fixed position during cephalometric radiography, a decrease in the distance of menton or the hyoid bone from the lower border of the film suggests an increase in the distance between the ear canal and the hyoid and lower border of the mandible.

#### Radiation protection features

**Figure 1:**
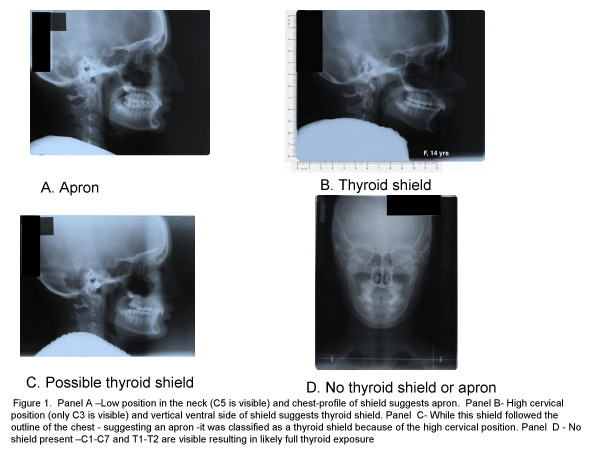


The radiation protection features that were abstracted from the cephalometric radiograph were the presence of a thyroid shield and a lead apron. When the superior border of the radio-opaque outline of a radiation protection garment did not follow the profile of the patient's chest, or when it protected the neck, a thyroid shield was said to be present. An apron was said to be present when the radio-opaque outline followed the patient's chest. When in doubt whether a patient was wearing a thyroid shield or an apron, a thyroid shield was considered to be present (see Figure 1).

#### Statistical analyses

The anatomical neck exposure characteristics were related to age and gender using generalized estimating equations. The prevalence of thyroid shields or aprons was determined by calendar year, age, and gender. The identity link and a normal error function were used for continuous outcomes and the logit link and a binomial error distribution were used for binary outcomes. The within-patient correlation of multiple radiographic observations within the same patient was taken into account by means of the generalized estimating equation methods [10]

## Results

### Patients

418 patient charts were randomly selected, 373 or 89% of which were included in the main analyses. Of the 45 non-included patient charts, 33 contained no dental cephalometric x-rays (8%), 11 (3%) could not be evaluated because the cephalometric X-ray was not in the chart or could not be assessed (e.g., overexposed film, processing error), and 1 chart had an undated cephalometric X-ray. Birth year and gender were similar among included and excluded patient charts. 79% (n = 295) of the 373 orthodontic patients were less than 20 years old and 60% (n = 225) were female. Among the 295 patients less than 20 years old, 55% were female (n = 162), and among the 78 patients older than 20, 81% (n = 63) were female. Records indicated that a total of 1212 cephalometric radiographs were taken in the 373 patients. Of these 1196 (99%) had a known exposure date, and 1190 (98%) could be assessed.

### Anatomical neck features

Among the 763 cephalometric radiographs with no shielding, chronological age was related to the amount of neck structure exposed, independent of gender. Between the age of 5 and 14, there was on average a decrease of 0.10 vertebral units for every one-year increase in age (standard error, 0.05). After the age of 14 there was no significant association between chronological age and the most inferior portion of the spine that was visible (regression slope, -0.01; p-value = 0.18). Chronological age was also related to the amount of film exposed below the inferior border of the hyoid. For every 1-year increase in age between the age of 5 and 14, the amount of film exposed decreased by 0.9 mm (95% confidence interval: 0.3 mm – 1.4 mm). This decrease in neck exposure with increasing age continued above the age of 14 where the amount of exposed film below the hyoid bone continued to decrease by 0.2 mm per year (95% confidence interval: 0.02 mm -0.3 mm.). There was no similar association between the distance of menton from the border of the X-ray and chronological age.

The amount of anterior neck structure exposed also depended on gender, independently of age. The number of vertebrae visible was independent of gender at any age.

Among patients 14 years and younger, the amount of film exposed inferior of the hyoid was 9 mm more in females than males (95% confidence interval: 6 mm to 12 mm). Among patients 15 years and older, the amount of film exposed apical of the hyoid bone was 2 mm more in females than males (95% confidence interval: 0.2 mm -3.8 mm). Among patients 14 years and younger, the amount of film exposed inferior of the menton was not different in females than males (3.4 mm difference; 95% confidence interval: -3.6 mm – 11.3 mm). Among patients 15 years and older, the amount of film exposed inferior to the menton was 5.1 mm more in females than males (95% confidence interval: 4.8 mm – 5.3 mm).

### Shielding

A thyroid shield was visible in 19.2% of the reviewed films (95% confidence interval: 16.2%-22.7%) (Table 1). Significant secular trends were present in thyroid shield visibility: 2.9% between 1973 and 1990, 47.9% between 1991 and 2000, and 10.4% between 2001 and 2003. These differences in thyroid shield visibility across three decades were highly significant (p < 0.0001). Both gender and age influenced the likelihood for the presence of a thyroid shield during some of these time periods. Between 1973 and 1990, the odds for seeing a thyroid shield was 5.3 higher (CI: 1.6–17.3) for adults than non-adults, and 3.0 times more common for males than females (CI: 0.9–9.6). Between 1991 and 2000, the odds for seeing a thyroid shield on the cephalometric radiograph were similar for adults and non-adults, and females were 1.8 times more likely to have a visible thyroid shield than males (CI:1.2–2.9). Between 2000 and 2003, there was no effect of gender, but adults were 9.6 times more likely to have a visible thyroid shield (CI: 1.4–64.0). An apron was visible in 16.6 % of the reviewed films (95% confidence interval: 13.9% -19.8%) (Table 2). Significant secular trends were present in apron utilization: 3.5% between 1973 and 1990; 47.9% between 1991 and 2000, and 72.7% between 2001 and 2003 (p-value < 0.0001).

The thyroid shield and the apron resulted in substantially different amounts of cervical exposure (Table 3). Among individuals wearing a thyroid shield or an apron, the most apical vertebrae that were typically exposed was C3 and C4 respectively. Among individuals not wearing shielding the most apical vertebra exposed was C5.

## Discussion

Findings indicate that in the absence of shielding, the amount of neck tissue exposed was larger in females than in males, and larger in children than in adults. Overall, usage of the thyroid shield occurred in less than 1 out of 5 cephalometric radiographs. The presence of a thyroid shield depended on calendar year, and during some periods both on the age and the gender of the patient. From 1973 until 1990, thyroid shields were used for less than 3% of the cephalometric X-rays. Thyroid shields were seen less often in children and females than in adults or males during this period. From 1991 until 2000, the prevalence of thyroid shield was 47%. No adult/child differences were observed during this period and females were more likely than males to wear thyroid shields. From 2001 until 2003, there was a drop in the thyroid shield utilization to 10% with a higher utilization in adults than children and no gender differences.

In the absence of a thyroid shield, the amount of neck structure radiated depends primarily on the orientation of the film, and less so on the age and the gender of the patient. The orientation, landscape or portrait, of the cephalometric film determines the amount of neck-tissue exposed to ionizing radiation (that is, if the beam is collimated to the film). If the long axis of the film is parallel to the spine (portrait orientation), thoracic vertebrae are typically exposed indicating a full exposure of the thyroid, and partial exposure of the esophagus. With a perpendicular orientation of the film to the spine (landscape orientation) only cervical vertebrae are typically exposed, and a fraction of the thyroid, as opposed to the whole thyroid, is exposed to ionizing radiation.

It has been reported that the soft tissues of the neck, including the thyroid, descend inferiorly with increasing age, but no data could be identified that describe this phenomenon [2]. The findings of this study suggest that children and females will have, regardless of film orientation, more neck structure exposed than adults or males. This finding may be because film sizes are constant, while the skull and neck of a children and females are smaller than those of adults and males. In addition to the amount of neck structure exposed in children, there is also evidence that the thyroid is positioned higher in the neck of children than of adults [2]. These findings suggest that neck structures are more likely to be exposed in those individuals who are most radiosensitive: children and females.

While the fraction of radiographs taken outside of the dental school could not be established, the observed secular changes in thyroid shield practices within this study may be partly explained by changes in radiological practices inside the dental school. Between 1973 and 1990 the cephalometric radiographs were taken either in the orthodontic clinic or outside. From 1991 and on, most cephalometric radiographs were taken in the oral radiology clinic. Most likely, the drastic increase in the utilization of thyroid shields coincided with the switch from taking radiographs in the orthodontic department to the oral radiology clinic. The drop in utilization from 2001 can partly be explained by the requirement to include at least the 3^rd ^cervical vertebra in the cephalometric radiographs to determine skeletal age [3,11-17]. In order to achieve this goal, the thyroid shield was not utilized for many patients or placed much lower, or only a leaded apron was used as a shield. The use of a thyroid shield on small children was difficult because of the thyroid shield design, as was reported in another study [18] suggesting why aprons were the most common sole radiation protection device after 2000.

The infrequent thyroid shield utilization may not be unique to the patients in this study. The previous manager of the X-ray control section of the Washington State Department of Health Radiation Protection has observed during surveys in the 1990s that thyroid shields are rarely used for intra-oral films (2%), and occasionally used for extra-oral x-rays (5%)[19]. A recent survey of routine cephalometric radiographs reported on the prevalence of thyroid and cricoid ossifications of laryngeal cartilages in children and adults[20] providing an indication of the potential general lack of use of thyroid shields in at least one other university setting. A review of 24 issues of the American Journal of Orthodontics (2003–2004) shows that less than 10% of the evaluable cephalometric X-rays had thyroid collars visible, a prevalence similar to that identified in this study. Current advertisements of cephalometric X-ray units [21,22], or for professional dental meetings [23], provide additional examples of cephalometric radiographs that do not reveal the presence of a thyroid shield. The extent to which publications in a professional journal, X-ray unit advertisements, or professional meeting advertisements reflect or influence clinical practice is not known. Nonetheless, it portrays a picture of a generalized lack of use of thyroid shields in cephalometrics.

Several surveys have reported low thyroid shield utilizations in dental settings, suggesting that the findings reported in this study are not limited to cephalometric radiography. One mail survey of 7940 dental offices in 1985 in Virginia and Florida had a 28% response rate and the findings suggested that thyroid collars were not routinely used in 83% of the offices [7] and not available in 74% of the offices. A mail survey of 398 dentists in 1992 in Michigan had a 67% response rate and indicated that thyroid collars were not used in 51% of the practices[8]. In a survey of 132 staff members within the department of dentistry of a teaching hospital, 40% reported not using a thyroid cover [7]. In contrast to these low utilization rates, a mail survey of North American schools with a 100% response rate in 2000 indicated that thyroid shields were consistently used for extra- and intra-oral radiography in 74% and 85% of the schools respectively [24]. Such self-reported statistics are open to overt selection and reporting biases.

In general, the decision when to use a thyroid shield is complex. For panoramic radiography, the most common extra-oral radiation exposure, there is a consensus that thyroid shields should not be used [25]. For cephalometric radiography, a thyroid shield may be contra-indicated, if skeletal age is to be determined based on cervical vertebrae. For bitewings, the most common type of radiograph used in dentistry, a shield has been reported to be cost-ineffective with rectangular collimation since it does not significantly reduce radiation to the thyroid [26, 27]. These complexities combined with the lack of unequivocal guidelines in the use of thyroid shielding for both intra- and extra-oral radiography indicate that clinical decisions on thyroid shield utilization can be confusing.

Weaknesses of this study include the limited regional nature of the data, the lack of information on the diagnostic goals of the clinicians, and the measurement error in determining the presence or absence of shielding. The data only reflect the radiation protection practices seen in patients presenting in the dental school. At best, the practices are reflective for the Seattle urban area. Minimal information was available related to the diagnostic purposes of the clinician. In particular, we did not abstract information from the charts to assess whether indeed skeletal age was determined based on cervical vertebral morphology. The absence of a visible thyroid shield or an apron on a cephalometric radiograph does not necessarily imply the absence of shielding. Possibly, the shield could have been placed outside of the scope of the film. Also, we do not have information to what extent thyroid shields were not used because it may have been thought to interfere with the imaging. Such a practice may be hinted in the increased utilization of aprons after 2000.

In conclusion, young individuals, who are more radiosensitive than adults, have more neck structure exposed in cephalometric radiography. In this survey, thyroid shields were less likely to be employed in children than in adults. These findings indicate that dose determination studies in *adult *phantoms where the neck structures are carefully *excluded *from any direct radiation may be unrepresentative of real-life doses to the neck in children without thyroid shielding. Estimation of real-life doses is important in providing a better understanding of the risks associated with radiation.

## Competing interests

The author(s) declare that they have no competing interests.

## Authors' contributions

Philippe Hujoel^1,2^: design, analysis and writing; abstraction of a limited number of charts

Lars Hollender^3^: Writing and interpretation of radiographs

Anne-Marie Bollen^6^: Provided orthodontic expertise with respect to chart content and abstraction

John D. Young^4^: primary data abstractor

Joana Cunha-Cruz^1,7^, assisting in data analysis

Molly McGee^5^: Writing and discussion of findings

Alex Grosso^5^: discussion of findings.

## Pre-publication history

The pre-publication history for this paper can be accessed here:



## Figures and Tables

**Table 1:** Prevalence of thyroid shield usage by decade, gender and patient's age

**Thyroid Shield**	**1973–1990**	**1991–2000**	**2001–2003**	**Overall**
**Overall**	2.9%	47.9%	10.4%	**19.2%**
**Gender**				
**Females**	1.9%	53.7%	12.8%	**16.3%**
**Males**	4.3%	38.7%	7.9%	**21.3%**
**Age group**				
**Adults (> 20 years old)**	7.3%	51.2%	42.9%	**24.8%**
**Children and adolescent**	1.8%	47.0%	7.1%	**17.9%**

**Table 2:** Prevalence of apron shield usage by decade, gender and patient's age.

**Apron**	**1973–1990**	**1991–2000**	**2001–2003**	**Overall**
**Overall**	3.5%	28.1%	72.7%	**16.6%**
**Gender**				
**Females**	2.2%	21.4%	79.5%	**13.4%**
**Males**	5.4%	38.7%	65.8%	**21.5%**
**Age group**				
**Adults (> 20 years old)**	4.4%	25.6%	57.1%	**13.9%**
**Children and adolescents**	3.2%	28.7%	74.3%	**17.3%**

**Table 3:** Association between type of radiation protection device (apron versus shield) and most inferior vertebra visible.

	**Thyroid Shield**	**Apron**	**No lead shield**
	N = 231 (19.2%)	N = 197 (16.6%)	N = 763 (64.1%)
*C1*	3 (1.3%)	-	1 (0.1%)
*C2*	31 (13.5%)	3 (1.5%)	4 (0.5%)
*C3*	163 (71.2%)	45 (22.7%)	27 (3.5%)
*C4*	30 (13.1%)	94 (44.8%)	112 (14.7%)
*C5*	2 (0.9%)	49 (24.8%)	306 (40.1%)
*C6*	-	5 (2.5%)	234 (30.7%)
*C7*	-	1 (0.5%)	57 (7.5%)
*T1*	-	1 (0.5%)	13 (1.1%)
*T2*	-	-	9 (0.8%)
